# Covid-19 Symptomatic Patients with Oral Lesions: Clinical and Histopathological Study on 123 Cases of the University Hospital Policlinic of Bari with a Purpose of a New Classification

**DOI:** 10.3390/jcm10040757

**Published:** 2021-02-13

**Authors:** Gianfranco Favia, Angela Tempesta, Giuseppe Barile, Nicola Brienza, Saverio Capodiferro, Maria Concetta Vestito, Lucilla Crudele, Vito Procacci, Giuseppe Ingravallo, Eugenio Maiorano, Luisa Limongelli

**Affiliations:** 1 Odontostomatology Unit, Department of Interdisciplinary Medicine, “Aldo Moro” University of Bari, 70124 Bari, Italy; gianfranco.favia@uniba.it (G.F.); angela.tempesta1989@gmail.com (A.T.); g.barile93@hotmail.it (G.B.); capodiferro.saverio@gmail.com (S.C.); 2 Covid-19 Intensive Care Unit, Department of Interdisciplinary Medicine, “Aldo Moro” University of Bari, 70124 Bari, Italy; nicola.brienza@uniba.it (N.B.); mconcetta.vestito@gmail.com (M.C.V.); 3 Covid-19 Emergency Unit, Department of Emergency and Urgency, University Hospital Policlinic of Bari, 70124 Bari, Italy; lucilla.crudele@uniba.it (L.C.); v.procacci59@gmail.com (V.P.); 4 Pathological Anatomy Unit, Department of Emergency and Organ Transplantation, University of Bari Aldo Moro, 70124 Bari, Italy; giuseppe.ingravallo@uniba.it (G.I.); eugenio.maiorano@uniba.it (E.M.)

**Keywords:** Covid-19, oral lesions, oral ulcers, classification

## Abstract

The aim of this study is to report on the oral lesions detected in 123 patients diagnosed at the University Hospital of Bari from October 2020 to December 2020, focusing on the correlation of clinical and pathological features in order to purpose a new classification. Methods. General and specialistic anamnesis were achieved and oral examination was performed. The following data were collected: age/gender, general symptoms and form of Covid-19, presence and features of taste disorders, day of appearance of the oral lesions, type and features of oral lesions and day of beginning of therapies. If ulcerative lesions did not heal, biopsy was performed. Results. Many types of oral lesions were found and classified into four groups considering the timing of appearance and the start of the therapies. Early lesions in the initial stages of Covid-19 before the start of therapies was observed in 65.9% of the patients. In the histopathological analysis of four early lesions, thrombosis of small and middle size vessels was always noticed with necrosis of superficial tissues. Conclusion. The presence of oral lesions in early stages of Covid-19 could represent an initial sign of peripheral thrombosis, a warning sign of possible evolution to severe illness. This suggests that anticoagulant therapies should start as soon as possible.

## 1. Introduction

Since the identification of the Severe Acute Respiratory Syndrome Coronavirus 2 (SARS-CoV-2) in December 2019 in Wuhan, China, millions of cases have been diagnosed worldwide, with mortality rates ranging from 3% to 12% [1,2]. The Centers for Disease Control and Prevention in the clinical course of the Coronavirus Disease 19 (Covid-19) distinguished the following forms: asymptomatic infection, mild, moderate, severe and critical illness [1]. In addition to fever, fatigue, dry cough, sore throat, breathing difficulties, pneumonia and respiratory complications that often deteriorate to severe acute respiratory syndrome, SARS-CoV-2 may cause plenty of other complications that involve the kidney, heart, central and peripheral nervous system and gastrointestinal tract [3]. Although gustatory dysfunctions as a clinical presentation of mild-to-moderate forms of Covid-19 were clearly stated in [4] in April 2020, to the best of our knowledge, Chaux-Bodard et al. described the first oral association with Covid-19 in a patient presenting an irregular ulcer on the dorsal side of the tongue [5]. After this report, many case reports followed. White plaques, several painful ulcers, non-specific nodules, severe geographic and fissured tongue, small blisters, petechiae, pustular enanthema, desquamative gingivitis, angina bullosa and erythema multiforme-like lesions were described with not well-known etiopathogenesis and unclear direct virus correlation [6,7,8,9,10,11]. While histopathological studies with a broad spectrum of skin dermatoses associated to Covid-19 were largely reported in the current English literature [12], histopathological analysis of oral mucous lesions is still not described.

The aim of this study is to report on the oral lesions detected in 123 patients diagnosed at the University Hospital of Bari from October 2020 to December 2020, focusing on the correlation of clinical and pathological features in order to purpose a new classification.

## 2. Materials and Methods

This study was carried out in accordance to the principles of the Declaration of Helsinki and approved by the Independent Ethical Committee active in the University of Bari, Italy (Study No. 4652, Prot. 66/CE); patients released informed consent for diagnostic and therapeutic procedures and for possible use of the biologic samples for research purposes.

Patients included in the current study followed these inclusion criteria:

(1) The presence of SARS-CoV-2 confirmed by reverse transcription polymerase chain reaction (RT-PCR) after nasal and oropharyngeal swabs;
(2) Adult patients hospitalized at the University Hospital Policlinic of Bari from October 2020 to December 2020.

Patients with certain pre-existing lesions, which means all those that were symptomatic of pre-existing systemic and local conditions previously diagnosed and well-known to the patients, as well as patients with traumatic lesions, were excluded. Because of their home self-confinement, people with the asymptomatic and mild forms were excluded too.

All the patients were visited by three oral pathologists properly dressed in personal protective equipment; those with moderate form were visited at the Emergency Unit of University Hospital Policlinic of Bari, while the others with severe and critical illness were visited at the Covid-19 Intensive Care Unit of the University Hospital Policlinic of Bari.

General and specialistic anamnesis were achieved from patients or derived from medical records if the patient was unable to communicate. The following data were collected: age and gender, general symptoms of Covid-19, form of Covid-19 according to WHO classification [1], presence of taste disorders, day of appearance of the oral lesions after the onset of general/systemic symptoms and day of beginning of therapies. With respect to taste disorders the authors distinguished real dysgeusia, hypogeusia and ageusia.

After the WHO eight-step intraoral examination, the following clinical data were collected: type of lesion and features (site, size and numbers), symptoms referred and quality of oral hygiene. Where necessary, cytological smears were collected by brushing and following 80% alcoholic spray fixation and stained with Papanicolaou stain and Periodic Acid Shiff (PAS).

The following topical treatments were administered:

1. Hyaluronic acid gel and chlorhexidine 2% mouthwash or gel (twice a day) for 14 days in patients with ulcero-erosive lesions [13,14];
2. Miconazole Nitrate twice a day in patients with cytological diagnosis of candidiasis [15];
3. Tranexamic acid for local hemorrhages [16].

If ulcerative lesions did not heal after 14 days in patients with the mild form of Covid-19, biopsy was performed under local anesthesia, with prior rinsing with chlorhexidine 2% mouthwash of at least one minute.

All surgical samples were promptly fixed in neutral-buffered formalin for 48 h and sent to the Pathological Anatomy Unit of the University of Bari, then embedded in paraffin, sectioned at 4 µm thickness and stained with hematoxylin-eosin (H&E).

Finally, lesions were classified into four groups:

1. Probably pre-existing conditions: all the para-physiological lesions endemic in the general population;
2. SARS-CoV-2-related lesions. The authors considered lesions as virus-related when they appeared together with general symptoms or within one week after the onset of general symptoms and always before the beginning of therapies;
3. Treatment-related lesions: lesions that appeared after the start of the Covid-19 specific therapies;
4. Lesions mainly related to poor oral hygiene.

All the diagnostic-therapeutic protocol was detailed in a flowchart (Figure S1).

## 3. Results

### 3.1. Patients Data

A total of 123 patients were enrolled in this study, 70 M and 53 F (M:F ratio 1,3:1) with a median age of 72 years. Patient data are summarized in Table 1.

### 3.2. Oral Manifestations

**Figure 1:**
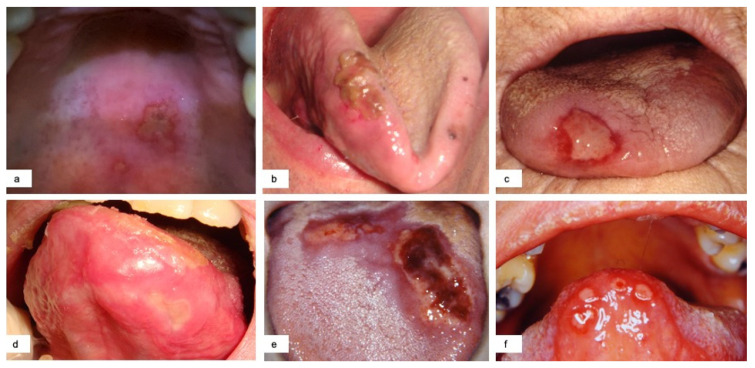
Single ulcerative lesions with diameter larger than 1 cm located in different sites (**a**,**b**) with associated petechiae (**c**). Multiple ulcerative lesions with erythema multiforme-like aspect and tendency to coalesce (**e**,**f**) and herpetiform-like aspect (**d**).

Ulcerative lesions (65–52.8%) were the most frequently detected injury. They often were painful and presented as single (40%) (Figure 1 a,b,c) or multiple (60%) lesions. The latter could occur with a herpetiform figured aspect (Figure 1d) or with a diffuse erythematous base with evident multiple apthoid necrotic lesions coalescing in larger ulcerative areas with yellowish fibrin covering, globally resembling erythema multiforme-like disease (Figure 1 e,f). When ulcers or larger necrotic areas involved the cutaneous side of the lips, they evolved in crusts. Only in four cases, in which the authors noticed a delay in healing (>14 days), biopsy was performed. These lesions were mainly located on the tongue, palate, lip and cheek. In seven cases, the ulcerative lesions were preceded by blisters as referred by patients. In 92% of the cases the ulcerative lesions appeared together with general symptoms or within one week after the onset of general symptoms, then defined as early.

**Figure 2:**
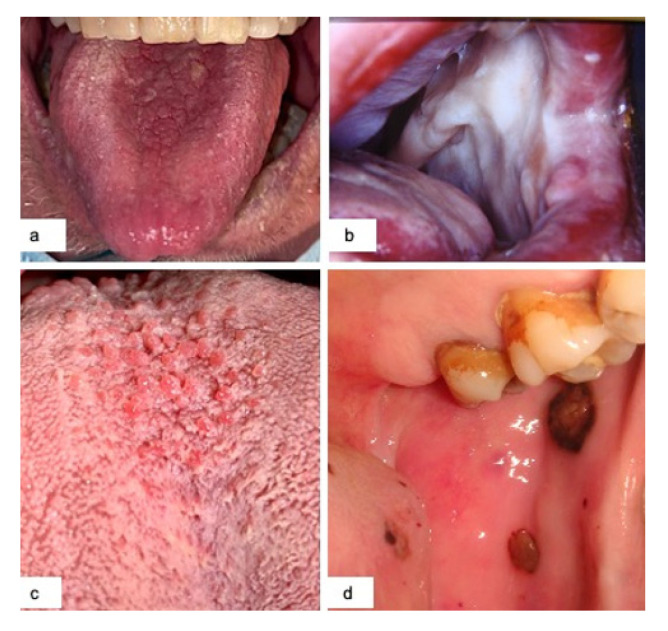
Red candidiasis median rhomboid glossitis-like (**a**); diffuse white candidiasis in an intubated patient (**b**); hyperplasia of the lingual papillae (**c**); angina bullosa of the cheek with associated petechiae (**d**).

Candidiasis was noticed in 28 patients (22.7%), most of which were red forms (21) located on the tongue, mainly with median rhomboid glossitis-like appearance (Figure 2a), and palate followed by white forms (7) (Figure 2b). The former were principally highlighted in the moderate forms of Covid-19, while the latter were often seen in intubated patients with severe and critical illness. The symptoms, if referred, associated with candidiasis were pain and overall burning. A good recovery of the lesions was observed with topical antifungal therapy (miconazole nitrate twice a day).

Blisters were detected in 19 patients (15.4%). They suddenly collapsed into superficial erythematous-ulcerative lesions and were mainly located on the tongue and palate; two cases were associated with pain and low bleeding. They were often seen within the first week after the onset of general symptoms.

Hyperplasia of papillae was highlighted in 48 cases (39%). It was always present in patients with taste disorders and appeared as red enlargement of the papillae both on the dorsum and on the sides of the tongue (Figure 2c).

Petechiae were noticed in 14 cases (11.4%). Frequently asymptomatic, they were located on the hard and soft palate and tongue. They mostly appeared after the start of therapies often in association with angina bullosa (Figure 2d).

Ulcero-necrotic gingivitis was unmasked in seven patients mainly in those with critical illness with poor oral hygiene due to the lack of teeth brushing. Gingivo-parodontal bleeding was often detected.

Angina bullosa was principally observed after the beginning of therapies, mainly appeared with brown-black single-multiple bullae, and it was located on the soft palate, tongue and cheek (Figure 2d).

Geographic tongue and fissured tongue occurred, respectively, in seven and five patients (5.6% and 4%). They were asymptomatic and probably pre-existing.

Spontaneous oral hemorrhage happened in only one patient (0.8%) with critical illness under anticoagulant therapy, and the bleeding derived from sublingual varices.

Considering the features of the lesions, the timing of presentations and the therapies administered, the lesions are classified in Table 2.

A total of 65.9% of the patients showed lesions definable as “Early”. This means that they appeared together with the onset of general symptoms or within one week and always before the beginning of Covid-19-specific therapies.

### 3.3. Taste Disorders

Dysfunction of the taste was noticed in all three groups of patients with a percentage higher than 80%. Particularly, real dysgeusia, intended as changes in taste discrimination (i.e., perception of bitter flavor rather than sweet or salty flavor), was noticed in 64% of patients, hypogeusia in 27% and ageusia in 9%.

Dysgeusia was always referred as one of the first symptoms.

### 3.4. Histopathological Analysis

From a histopathological point of view, it is possible to highlight different features depending on whether the analyzed part of the ulcer is central or peripheral.

**Figure 3:**
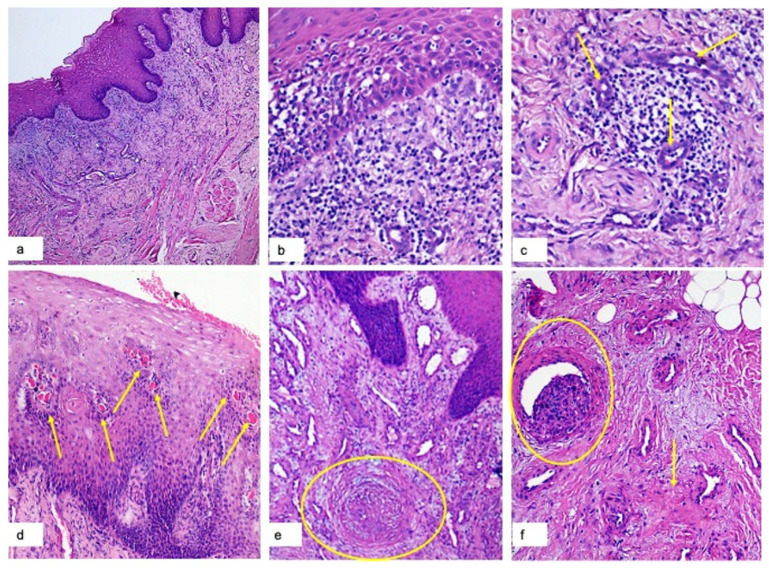
H&E Magnification 40×: superficial band-like lichenoid appearance of the inflammatory infiltrate with prominent vascular hyperplasia (**a**); H&E Magnification 150×: lympho-monocytes sub-epithelial infiltrate and edema, keratinocyte necrosis, and activation of Langerhans cells (**b**); H&E Magnification 180×: lympho-monocellular perivascular infiltration (**c**); H&E magnification 100×: Presence of multiple micro-thrombi (yellow arrows) of sub-epithelial small vessels (**d**); H&E Magnification 120×: total occlusion of a middle-size sub-epithelial vessel with initial organization of the thrombus (**e**); H&E Magnification 140×: partial occlusion of a deep vessel (yellow circle) and perivascular secondary fibrosis (**f**).

In the central necrotic-ulcerated parts, it denotes the complete lack of epithelial covering with a superficial layer of fibrin-enclosed basophilic debris (from alimentary and tissue origin), and microorganisms cover underlying sub-epithelial tissues showing prominent vascular hyperplasia (Figure 3a), perivascular hemorrhage and lymphomonocytes infiltration. In the deeper tissues this infiltrate has three main patterns: (1) superficial band-like lichenoid appearance (Figure 3a,b); (2) perivascular often with dense sleeve-like pattern (Figure 3c) (around small and medium size vascular structures); (3) peri-glandular, around lobules of minor salivary glands. Thrombotic vascular occlusion of small and medium size vascular structures is a frequent feature, in small vessels mainly with total occlusion and in larger vessels with partial occlusion (Figure 3d,e,f).

In the peripheral parts, corresponding to the epithelial covered border of the ulcers, the histopathological analysis revealed epithelial lesions: spongiosis, edema, leukocytosis, necrotic keratinocytes and presence of activated Langerhans cells (Figure 3e); tongue papillae showed a global hyperplastic aspect with lymphomonocytic infiltration and vascular hyperplasia.

## 4. Discussion

The coronavirus 2019 disease is a viral infection with multiorgan manifestations and variable severity of complications [11]. Although prevalence of dermatological manifestation is already reported [17], the prevalence of oral lesions is still unknown probably because oral cavity is not examined in Covid-19 patients. Since Chaux-Bodard et al. [5] described the first oral manifestation associated with Covid-19, many reports were published with plenty of lesions described. The most frequently detected oral manifestation is ulcer (54.1%) [18], although white plaque, severe geographic and fixtured tongue, petechiae, nodules, reddish macules, angina bullosa, blisters, necrotizing periodontal disease and erythema multiforme-like lesions were also described [19,20,21,22]. In this study, most detected lesions were ulcers (52.8%), both single and often multiple, with a tendency to merge into large necrotic areas. The blisters were detected in 15.4% of cases and referred in anamnesis in about 10% of patients with early ulcerative lesions. Different types of candidiasis were also diagnosed in a pool of 28 patients.

Seirafianpour et al. [23], in a systematic review of dermatologic Covid-19 disorder, applied an interesting distinguishment of skin lesions into virus-related lesions, virus-treatment related lesions and pre-existing lesions that could be exasperated by Covid-19 infection.

Along the lines of Seirafianpour et al., the authors decided to categorize lesions into four groups considering the following factors: the possibility they were pre-existing, time of onset of oral lesion compared to the appearance of general symptoms and start of therapy, quality of oral hygiene, and probably plaque-related conditions. The first group comprised “Probably pre-existing lesions”, which means conditions underestimated by patients, but in all likelihood already present at the moment of Covid-19 diagnosis. Indeed, geographic tongue and fissured tongue have a varied prevalence in the general adult population up to about 13% and 30%, respectively, and were often asymptomatic and underestimated by patients [24]. Considering the high prevalence in the general adult population, the fact that they were asymptomatic and not well known by the patients, the authors decided to insert geographic and fissured tongue in the first group.

The second group included “SARS-CoV-2-related lesions”. The authors decided to enclose in this group all the lesions that appeared together with the general symptoms within one week and always before the start of Covid-19-specific therapies. Considering this definition, some type of lesions, such as ulcerative ones or petechiae, were distinguished in early and late stages and allocated in different groups. The early petechiae were associated with vasculitis caused by SARS-CoV-19. The “Early” lesions belonging to this group were detected in 65.9% of the patients.

The third group included treatment-related lesions, which means all the lesions that appeared after the beginning of therapy and that were also related to multiorgan failures in several critical cases. This group enclosed late petechiae and angina bullosa mainly related to anticoagulant therapies and candidiasis related to corticosteroid and antibiotic combined therapies.

Finally, the fourth group included lesions associated with poor oral hygiene quality. Indeed, although intubated patients received mouthwash with a solution filled with chlorhexidine (2%, three times a day), the lack of mechanical teeth brushing caused the presence of abundant plaque underlying the ulcero-necrotic gingivitis.

In view of the above, the authors primarily considered the lesions of the second group. Four patients with early primary large necrotic area (enclosed in group 2) that did not heal within 14 days underwent biopsy in order to understand the physio-pathological mechanism underlying the formation of such big ulcers in an early stage of Covid-19.

Gianotti et al. [12] described the histopathology of skin Covid-19 lesions with a spectrum of findings that ranged from mild spongiosis of the epithelial layer to the important situation of vasculitis and extravasation of red blood cells.

To the best of our knowledge, this is the first study that widely describes the histological aspect of oral SARS-CoV-2- related lesions. The principal phenomenon that presents both in peripheral and central parts of “Early” oral necrotic areas is the thrombotic vascular occlusion of small and medium size vascular structures. Where the occlusion is complete or almost complete, necrosis of the superficial layers occurs with a wide inflammatory reaction in the deeper tissues organized in three different types of infiltration: (1) superficial band-like lichenoid appearance; (2) perivascular; (3) peri-glandular. At the periphery, where partial occlusion is observed, it is possible to denote signs of epithelial suffering with spongiosis, edema, leukocytosis, necrotic keratinocytes and activation of Langerhans cells with vascular hyperplasia in the deeper tissue.

Chemosensitive disorders, both olfactory and gustatory, emerged as highly prevalent symptoms during Covid-19. The clinical onset of chemosensitive disorders occurs characteristically in the very early stages of the symptomatic infection, generally in the first three days. The pathophysiological mechanisms leading to the olfactory and gustatory dysfunctions in COVID-19 infection are still unknown [25]. Two main theories were discussed in the current Covid-19-related literature. On the one hand, it is possible that viruses could infect peripheral neurons using the cell machinery of active transport to access the central nervous system [4], and on the other hand, inhibition of the ACE-2 receptor is possible. In fact, it is well known that ACE2 inhibitors can induce ageusia with a complex mechanism that involves G-protein-coupled protein and sodium channel present in the taste buds. SARS-CoV-2, infecting the cells and binding these receptors, could inactivate the latter, blocking the transformation of chemical gustatory signals into action potential and consequently the sensory perception of taste [25].

In this study, dysfunction of the taste was noticed in all three groups of patients with a percentage higher than 80%, and it was always referred as one of the first symptoms. The authors preferred to use the term taste dysfunction instead of dysgeusia because the spectrum of taste alterations was broad. In 64% of the patients with taste dysfunction real dysgeusia was noticed, intended as the changes in taste discrimination (i.e., perception of bitter flavor rather than sweet or salty flavor). Hypogeusia and ageusia, intended as reduction or complete lack of taste, were less frequent and, respectively, highlighted in 27% and 9%.

This research is, however, subject to potential limitations. This is an observational descriptive study with a brief follow-up of the patients. The authors noticed the lack of previous research studies involving a big sample like that described in this article. In fact, articles about oral lesions in Covid-19 patients published in the current literature are case reports or small series. Moreover, the collection of data for those patients intubated was achieved from medical records and not directly from the patients, so it is possible that some data are not complete.

Surely, further studies should be conducted, better if multicenter, in order to reduce these limitations at most.

## 5. Conclusions

This study on a large series highlighted that oral lesions in more than half of cases (65.9%) occurred in the early stage of Covid-19 before the beginning of specific therapies. Moreover, this study has unearthed that the physio-pathological mechanism, underlying the formation of early oral lesions, is the thrombosis of sub-epithelial and deeper vessels. Therefore, the presence of oral lesions could represent an initial sign of peripheral thrombosis, a warning sign of possible evolution to severe illness. This suggests that anticoagulant therapies should be started as soon as possible.

## Figures and Tables

**Table 1:** Patient data.

Form of Covid-19	N. of Patients	Age *(*Mean Value)	General Symptoms	Type of Oral Lesions	Oral Symptoms	Day of Appearance	Taste Disorders
**MODERATE**	95 (77%)	63	Fever > 39 °C, anosmia, cough, sore throat, congestion and runny nose, nausea or vomiting, muscle and body aches, dermatologic manifestation, pneumonia	- Geographic tongue (5) - Fissured tongue (4) - Ulcerative lesion (51) - Blisters (14) - Hyperplasia of papillae (33) - Angina bullosa (8) - Candidiasis (18) - Ulcero-necrotic gingivitis (1) - Petechiae (4)	- Pain - Burning - Bleeding - Difficulty to chewing and swallow	- T *: 25 (26.4%) - W **: 39 (41%) - Z ***: 31 (32.6%)	87%
**SEVERE**	21 (17%)	74	Fever >39 °C, anosmia, cough, sore throat, congestion and runny nose, nausea or vomiting, muscle and body aches, dermatologic manifestation, severe pneumonia, Dyspnea and hypoxia (SpO2 < 90%); severe respiratory distress	- Geographic tongue (2) - Fissured tongue (1) - Ulcerative lesion (11) - Blisters (5) - Hyperplasia of papillae (13) - Angina bullosa (2) - Candidiasis (4) - Ulcero-necrotic gingivitis (2) - Petechiae (6)	- Pain - Burning - Bleeding - Difficulty to chewing and swallow	- T *: 4 (19%) - W **: 11 (52.4%) - Z ***: 6 (28.6%)	88%
**CRITICAL**	8 (6%)	81	Acute respiratory distress syndrome, multiorgan failure	- Ulcerative lesion (3) - Hyperplasia of papillae (2) - Angina bullosa (1) - Candidiasis (6) - Ulcero-necrotic gingivitis (4) - Petechiae (4) - Spontaneous oral hemorrhage (1)	Not possible to achieve	- T *: 1 - W **: 1 - Z ***: 6	83%

* T: oral lesions that appeared together with the onset of general symptoms. ** W: oral lesions that appeared within one week after the onset of general symptoms and before Covid-19-specific therapies. *** Z: oral lesions that appeared after one week of the onset of general symptoms or after therapies.

**Table 2:** Classification of lesions.

Probably Pre-Existing Conditions	Geographic Tongue, Fissured Tongue
Sars-CoV-2-related lesions	Early ulcerative lesions, blisters, early erythema multiforme-like lesions, petechiae
Treatment-related lesions	Late ulcerative lesions, late erythema multiforme-like lesions, candidiasis, angina bullosa, spontaneous oral hemorrhage, petechiae
Lesions related to poor oral hygiene	Ulcero-necrotic gingivitis

## Supplementary Materials

The following are available online at https://www.mdpi.com/2077-0383/10/4/757/s1, Figure S1: FC1. Diagnostic therapeutic protocol of oral lesions in Covid 19 patients.
