# Supplementary figures and images for: Covid-19 Symptomatic Patients with Oral Lesions: Clinical and Histopathological Study on 123 Cases of the University Hospital Policlinic of Bari with a Purpose of a New Classification
Source: J Clin Med. 2021 Feb 13;10(4):757. doi: 10.3390/jcm10040757 (PMC7918830; doi:10.3390/jcm10040757)

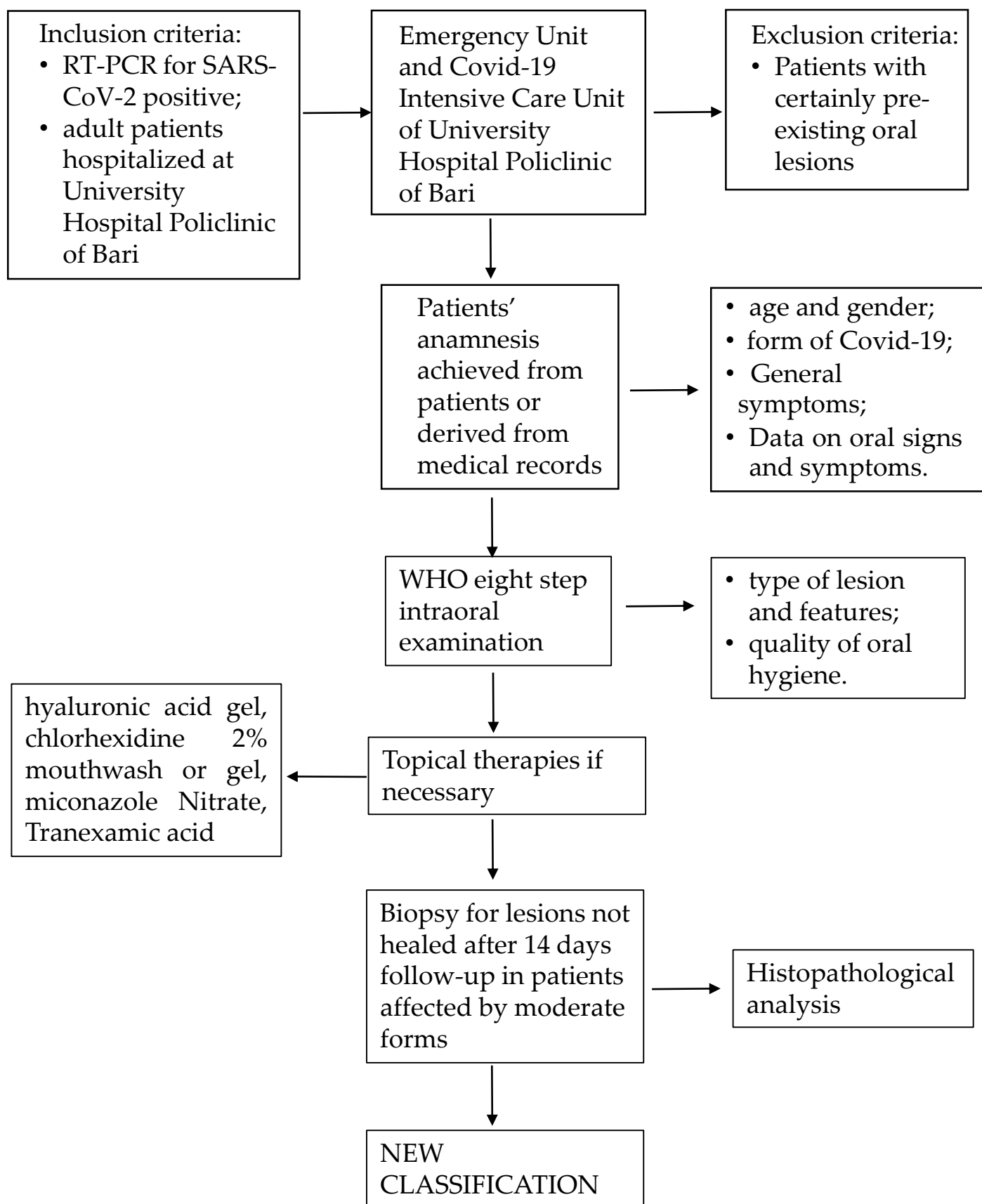

FC1. Diagnostic-therapeutic protocol of oral lesions in Covid-19 patients.

Supplement: Supplementary file 1 [file jcm-10-00757-s001.pdf]
